# Development of Auer bodies from giant inclusions associated with rough endoplasmic reticulum in acute promyelocytic leukemia

**DOI:** 10.1097/BS9.0000000000000145

**Published:** 2022-12-27

**Authors:** Yong-Xin Ru, Shu-Xu Dong, Jing Liu, Brian Eyden

**Affiliations:** a State Key Laboratory of Experimental Hematology, National Clinical Research Center for Blood Diseases, Haihe Laboratory of the Cell Ecosystem, Institute of Hematology and Blood Diseases Hospital, Chinese Academy of Medical Sciences & Peking Union Medical College, Tianjin, China; b Formerly Department of Histopathology, Christie NHS Trust, Manchester, United Kingdom

**Keywords:** Auer body, Giant inclusion, Primary granule, Promyeloblasts, Rough endoplasmic reticulum

## Abstract

Giant inclusions and Auer bodies in promyeloblasts were investigated in a study which included transmission electron microscopy (TEM) for morphology and ultrastructural cytochemistry for myeloperoxidase in 10 patients with acute promyelocytic leukemia (APL). Ultrastructural cytochemistry demonstrated positive myeloperoxidase reactivity in giant inclusions, expanded rER cisternae, Auer bodies and primary granules. TEM revealed that giant inclusions were adorned by degenerated rER membrane, some of them sharing features with Auer bodies. We hypothesize a novel origin for Auer body development in promyeloblasts of APL, namely that they originate from peroxidase-positive and expanded rER cisternae, and that primary granules were directly released from these expanded rER elements, bypassing the Golgi apparatus.

## 1. INTRODUCTION

Auer bodies were first described by light microscopy as stick-like and spiculate bodies in the cytoplasm of leukemic cells by John Auer in 1906.^1^ Since then, numerous ultrastructural observations have demonstrated a fibrillar or crystallized substructure to this inclusion in acute leukemias and granulocytic sarcomas.^2–4^ Many investigations have revealed that Auer bodies are mostly found in acute myelogenous leukemia (AML), especially in acute promyelocytic leukemia (APL) with large numbers in the cytoplasm of leukemic cells.^5,6^ Recently, clinical investigations have indicated that raised numbers of Auer bodies and primary granules in neoplastic cells were associated with severe hemorrhage and disseminated intravascular coagulation in APL patients.^7^

Histochemical studies have demonstrated that Auer bodies contain oxidase, myeloperoxidase, phosphatase and stain with periodic acid-Schiff (PAS), and Sudan black. They are negative for lipase, glycogen and deoxyribonucleic acid.^8^ Combined morphological transmission electron microscopy (TEM) and ultrastructural cytochemistry have confirmed Auer bodies as containing acid phosphatase and myeloperoxidase.^9^ Given that the characteristics of Auer bodies are the same as those of primary granules and azurophilic granules in normal granulocytes, some researchers have presumed that Auer rods in APL might originate from the fusion of azurophilic granules.^10^

Here, we demonstrate the presence of large cytoplasmic bodies, which we refer to as *giant inclusions*, and which coexist with typical Auer bodies in the leukemic blasts in APL. The morphologic characteristics and distinctive substructure of these giant inclusions suggest an alternative origin for Auer bodies in the cells of APL.

## 2. MATERIALS AND METHODS

### 2.1. Clinical data and laboratory examination

Ten previously untreated APL patients were referred to the Blood Diseases Hospital, Tianjin, between 2012 and 2022. They included 8 males and 2 females, aged between 10 and 50 years old. All patients were subjected to light microscope morphology, flow cytometry, cytogenetic analysis and molecular biological characterization, as well as TEM and ultrastructural cytochemistry. All patients were characterized by hypercellularity of myeloid blasts, the cytogenetic abnormality t (15; 17) (q24; q21) and positivity of PML/RAR. Diagnoses were made on the basis of the World Health Organization classification of myeloid neoplasms and acute leukemia.^11^ For comparison, mononuclear cells from bone-marrow aspirates of anemic patients were observed as control cells for the leukemic cells of APL.

### 2.2. Wright-Giemsa and cytochemical stains

Bone-marrow aspirate smears were stained with Wright-Giemsa and cytochemical stains, which included Sudan black, myeloperoxidase, PAS staining, alpha-naphthyl butyrate esterase (α-NBE), and naphthol AS-D acetate esterase (AS-D NAE).

### 2.3. Transmission electron microscopy

A portion of the mononuclear cells from the bone-marrow aspirates was conventionally fixed and embedded in resin. Briefly, the samples were fixed in 2.5% glutaraldehyde, postfixed in 1% osmium tetroxide, washed in phosphate-buffered saline, dehydrated in graded alcohols and embedded in Epon 812. Ultrathin sections at 60 nm were cut and stained with uranyl acetate and lead citrate. For detection of myeloperoxidase activity, mononuclear cells were incubated for 1 hour in Graham and Karnovsky medium, and then processed for electron microscopy as described above, and unstained sections observed by TEM.^12,13^

## 3. RESULTS

### 3.1. Wright-Giemsa stain and cytochemistry for myeloperoxidase

**Figure 1.**
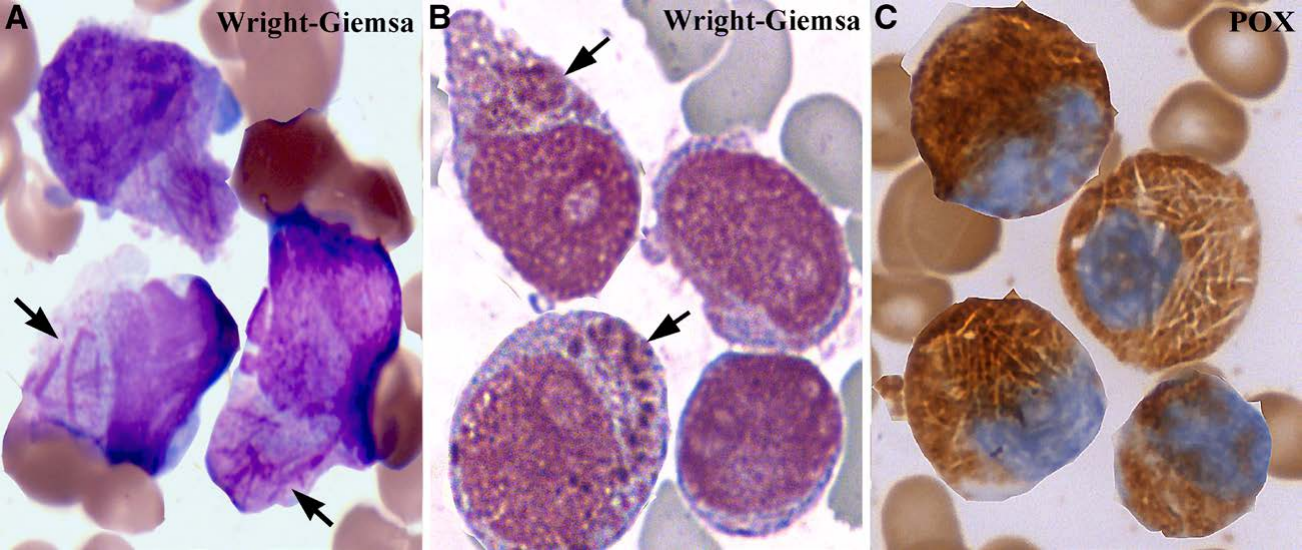
Wright-Giemsa and peroxidase staining of promyeloblasts. (A) Wright-Giemsa stain shows Auer rods (arrows) in the cytoplasm; (B) giant inclusions in a promyeloblast (arrows) in the cytoplasm; and (C) myeloperoxidase stain shows relatively unstained rod-like structures in APL promyeloblasts. APL = acute promyelocytic leukemia.

The Wright-Giemsa stain exhibited hyper-proliferation of promyelocytes in bone-marrow smears in all cases. All promyelocytes contained numerous round azurophilic granules, but a small proportion of cells included rod-like Auer bodies and irregularly shaped inclusions (Fig. 1A and B). The Auer bodies, inclusions and granules showed strong reactivity for myeloperoxidase (Fig. 1C).

### 3.2. General features of promyelocytes

**Figure 2.**
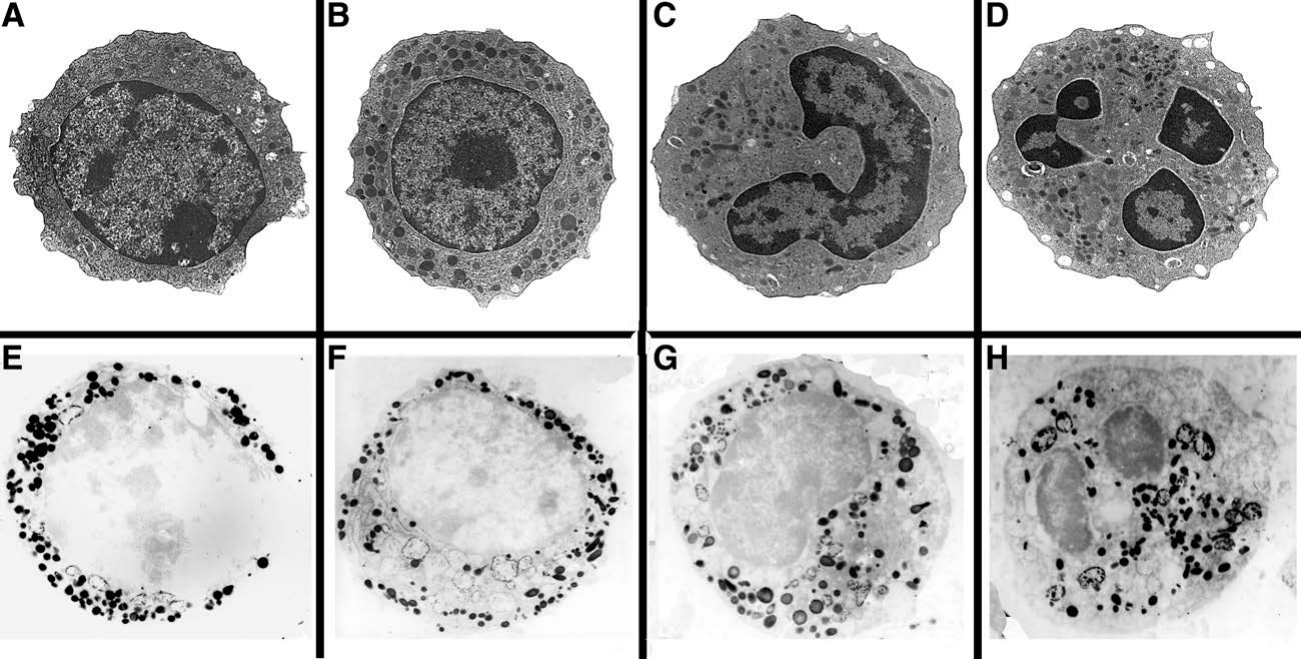
Normal neutrophils in (A)–(D), showing developmental stages of myeloblasts: myeloblasts (A), promyelocytes (B), myelocytes (C) to segmented granulocytes (D), ie, the mature neutrophils, ×5k. (E)–(H) Positive myeloperoxidase reactivity of primary granules of the neutrophil stages from myeloblasts, promyelocytes. myelocytes to segmented granulocytes respectively, ×5k.

Normally, neutrophils included slender profiles of rough endoplasmic reticulum (rER), primary granules in promyelocytes, and specific granules in myelocytes to segmented granulocytes; few of the above organelles are found in myeloblasts. There were no Auer bodies, irregularly shaped inclusions or expanded rER cisternae by morphological TEM or ultrastructural myeloperoxidase staining (Fig. 2).

**Figure 3.**
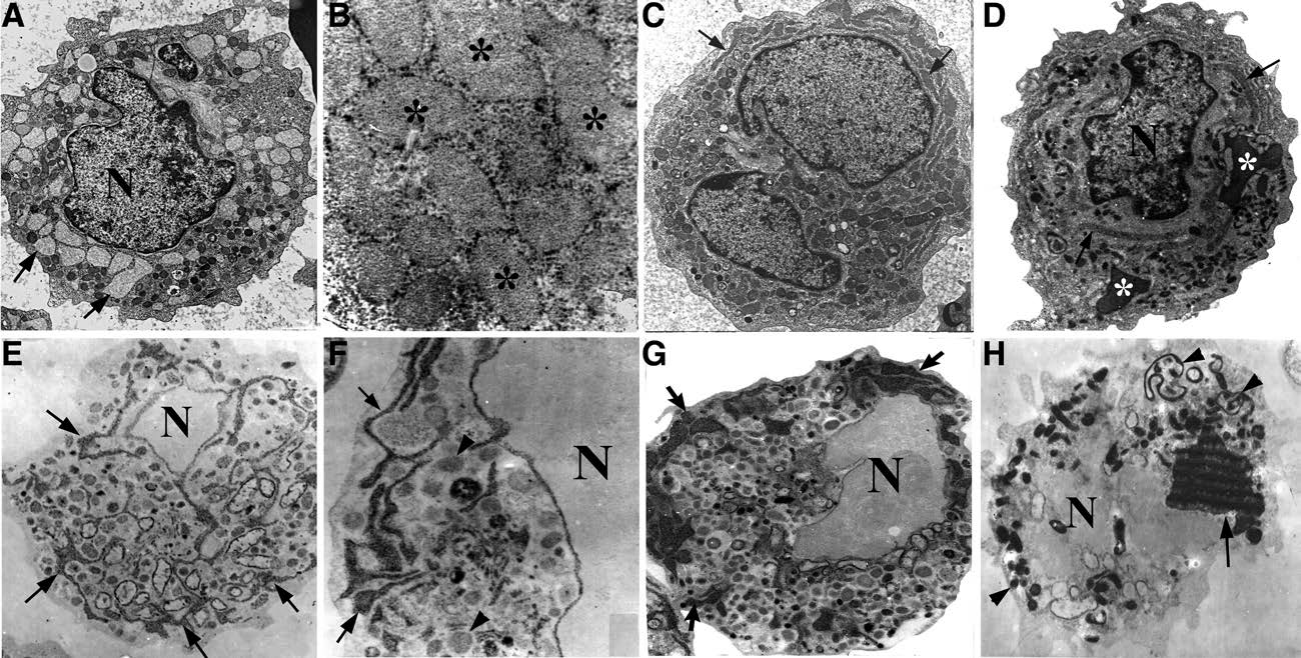
Expansion of rER in promyeloblasts and promyelocytes. (A) A promyelocyte full of pale-staining expanded rER cisternae (arrows), ×5k; (B) a high-magnification image of expanded rER cisternae with loosely textured content (*) and attached ribosomes, ×50k; (C) the expanded rER full of amorphous matrix (arrows) in a promyeloblast, ×5k; (D) linearly shaped dense rER-structures (arrows) and 2 inclusions also containing dense material (*), ×5k; (E) myeloperoxidase reactivity of rER cisternae (arrows) in a promyeloblast, ×5k; (F) a high-magnification image of rER-structures (arrows) and primary granules (arrowheads), ×50k; (G) strong myeloperoxidase reactivity of rER cisternae (arrows) at the cell periphery, ×5k; and (H) a giant, almost-rectilinear inclusion(arrow) and some primary granules in cisternae (arrowheads) positive for myeloperoxidase, ×5k. rER = rough endoplasmic reticulum.

In contrast, in most of the promyeloblasts in APL, rER cisternae were noticeably expanded and filled with a homogeneous matrix, so that some of them looked like irregular lakes in the cytoplasm. Primary granules varied in size and electron density, whereas specific granules were seldom found in promyeloblasts. Sometimes, structures resembling a simple vesicle, with a clear unstructured content, were observed and it was difficult to know whether these structures were elements of rER or a variant of primary granules (Fig. 3A–D).

The ultrastructural myeloperoxidase stain exhibited distinct reactivity of the amorphous matrix within expanded rER cisternae, the numerous primary granules, as well as lake-like rER elements and giant inclusions in APL promyeloblasts (Fig. 3E–H).

### 3.3. Giant inclusions and lake-like rER elements

**Figure 4.**
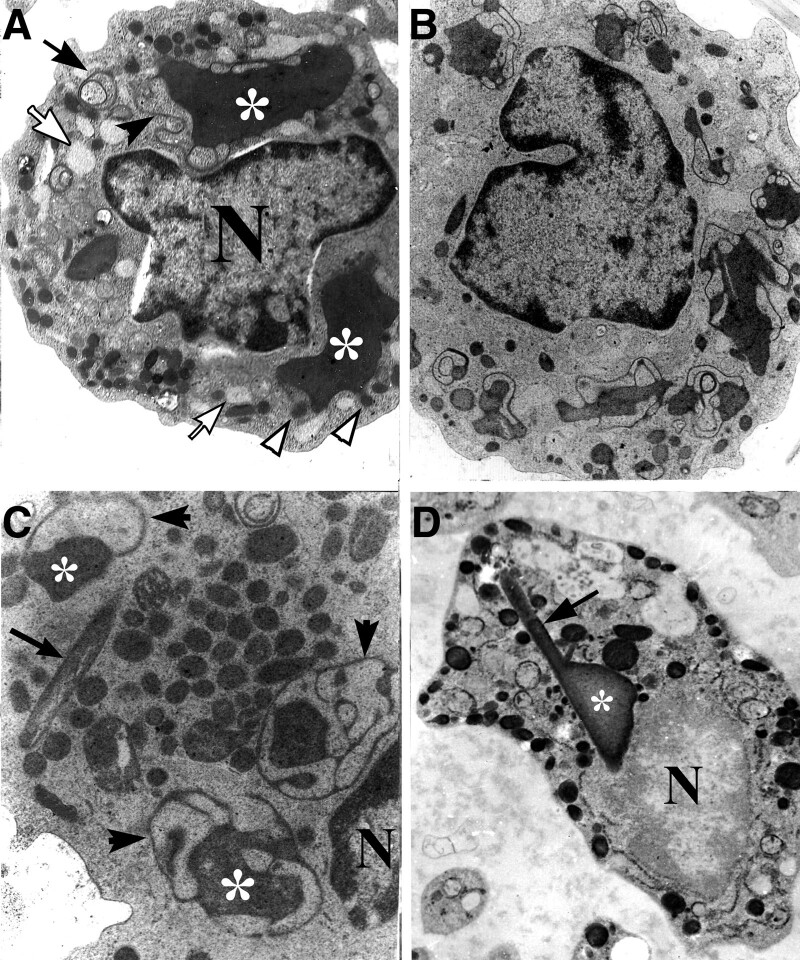
Giant inclusions associated with degenerated membrane. (A) Expanded rER (white arrows), degenerated membrane (black arrow) associated with giant inclusions containing homogeneous dense matrices (*) and primary granules (white arrowheads) budding off the inclusions, ×10k; (B) several giant inclusions adorned with membrane (arrows) giving a lace-like appearance, expanded rER (arrowheads) and small dense primary granules in a promyelocyte, ×10k; (C) giant inclusions (*) connected to surrounding membranes giving a lace-like appearance (arrowheads) and a typical Auer body (arrow) in a promyelocyte, ×10k; and (D) myeloperoxidase reactivity of a giant inclusion (*) with a rod-like handle (arrow) and positively staining primary granules, ×5k. rER = rough endoplasmic reticulum.

In the promyeloblasts of APL on TEM (Fig. 4A and B), all the giant inclusions containing a dense homogeneous matrix were surrounded by loops of membrane, giving the bodies a lace-like appearance. These loops of membrane were of uniform thickness—10 nm—and were dense as a result of enclosing the same material as in the central mass of the body. Auer bodies with typical features often coexisted with the giant dense inclusions in these promyeloblasts (Fig. 4C). All of the giant dense inclusions, Auer bodies, rER cisternae, and primary granules had strong reactivity to myeloperoxidase stain (Fig. 4D).

### 3.4. Pro-Auer bodies and primary granules

**Figure 5.**
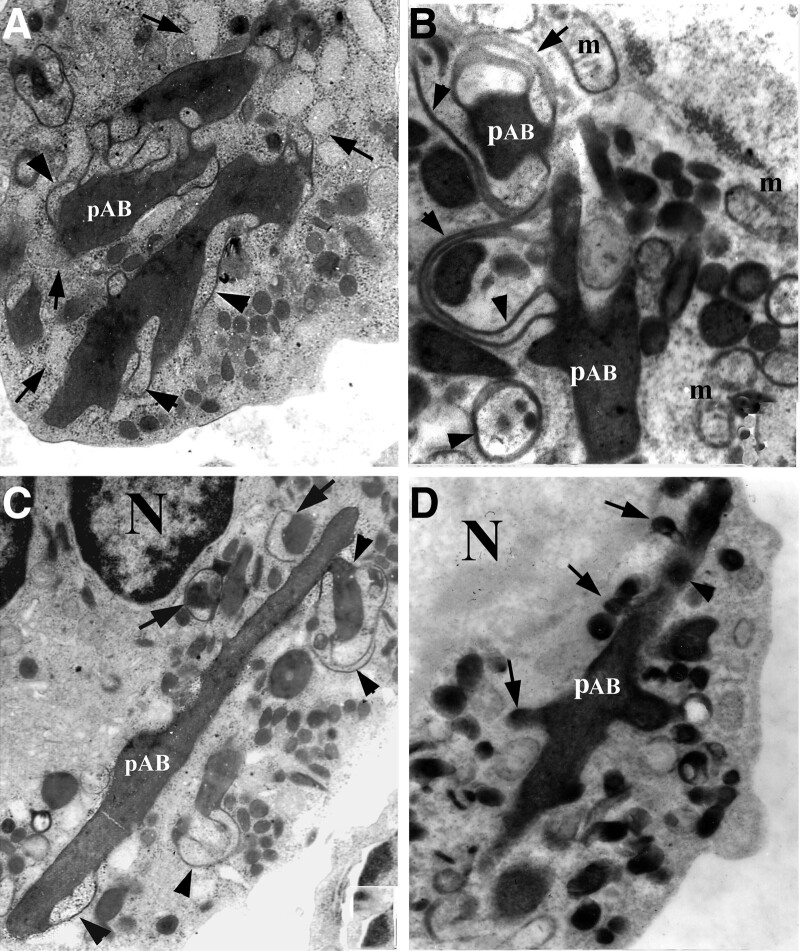
Transformation of giant inclusions to pro-Auer bodies. (A) Giant inclusions surrounded by degenerated membrane (arrowheads) in close association with rER (arrows), ×10k; (B) myeloperoxidase stain shows continuities between a pAB and elements of dense membrane (arrowheads), m, mitochondria, ×15k; (C) a rod-like pro-Auer body with a reduced amount of dense membrane (arrowheads) and smaller inclusions (arrows), ×1k; and (D) myeloperoxidase stain shows primary granules with the appearance of budding off a pAB (arrows) and a primary granule located within the pAB itself (arrowhead). N = nucleus, pAB = pro-Auer body.

Some giant inclusions had features in common with Auer bodies in having a rod-like main body like Auer bodies, but retaining the lace-like dense membrane profiles like those around the dense giant inclusions (Fig. 5A–D). We refer to this kind of inclusion as a “pro-Auer body.” The pro-Auer bodies often had attached smaller loops of dense membrane than those around giant inclusions, but no fine lamellar texture or crystal in the center like those in typical Auer bodies (Fig. 5A–C).

**Figure 6.**
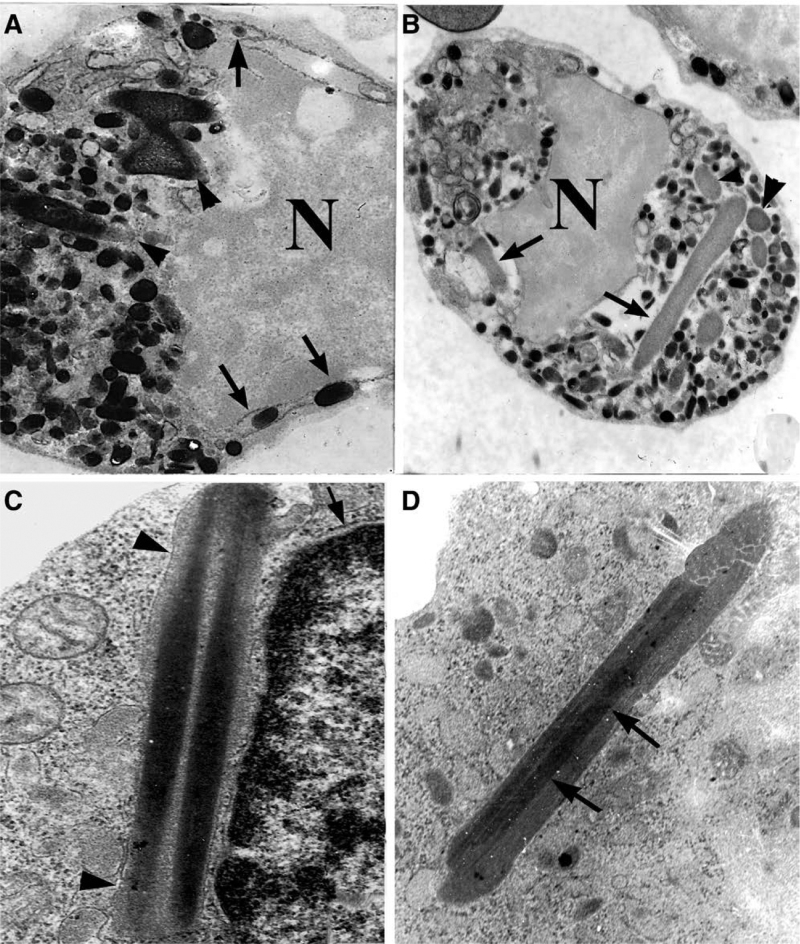
Mature Auer bodies. (a) and (b) show myeloperoxidase reactivity of promyeloblasts. (a) illustrates Auer bodies (arrowheads) – one of them irregular in shape – and granules in the perinuclear space (arrows), N, nucleus (unstained) ×10k; (b) shows longitudinal sections (arrows) and cross-sections (arrowheads) of Auer bodies in promyeloblasts, ×5k; (c), an Auer body has a double-crystalized center and an enveloping membrane (arrowhead) similar to that of the nuclear membrane (arrow), ×15 k; (d), an Auer body with a single crystallized center (arrows), ×15 k.

There were prominent primary granules with myeloperoxidase reactivity in the cytoplasm of promyeloblasts; some of them were budding out from pro-Auer bodies and giant inclusions (Fig. 5D), and some of them were located within perinuclear spaces and expanded rER cisternae (Figs. 3H and 6A).

### 3.5. Substructure of Auer bodies

**Figure 7.**
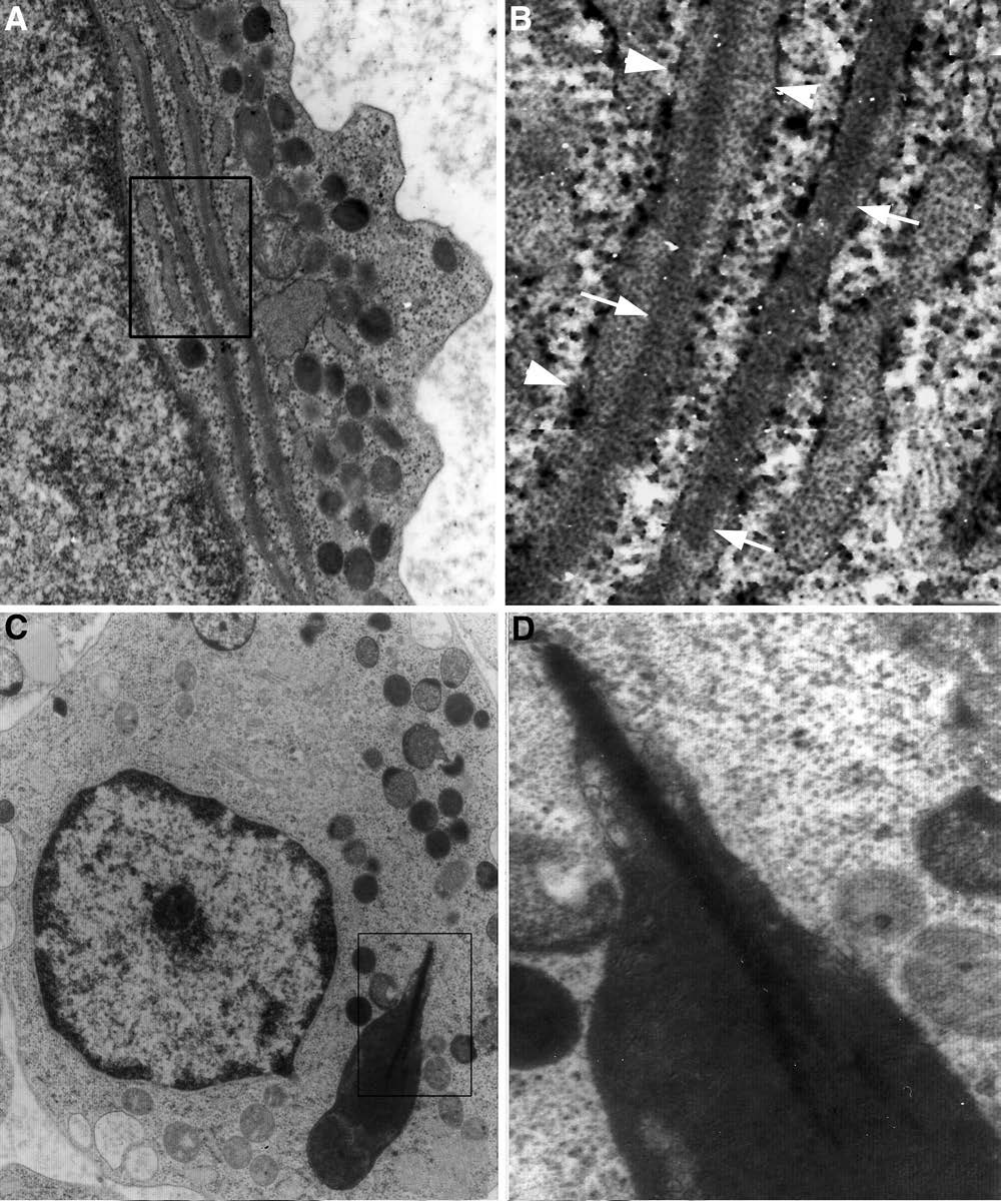
Early and late stages during Auer body formation. (A) a promyeloblast containing elongated profiles of rER, ×10k; (B) detail from (A) shows condensation of homogeneous material in rER (arrowheads) and formation of intracisternal rod-like structure (arrows), ×40k; (C) giant inclusion in a promyeloblast, ×6k; and (D) detail from (C) shows a dense rod-like structure in the center of the giant inclusion (arrowhead), a putative precursor for the Auer body, ×40k. rER = rough endoplasmic reticulum.

Auer bodies were often needle or rod-shaped, and had a smooth surface. Some of them contained homogeneous content, while others showed a fine lamellar or crystal-like inclusion (Fig. 6B–D). Crystalline inclusions, which may be precursors of the crystalline elements in Auer bodies, are present within some rER cisternae (Fig. 7).

## 4. DISCUSSION

Following the description in acute leukemia by Auer^1^ of the bodies named after him as Auer bodies or Auer rods, having a splinter-like appearance and tubular substructure on Wright’s stain by light microscopy, various pink-staining inclusions and granules occurring in leukemic blasts and nonleukemic cells have been reported.^14^ Some inclusions had a tubular substructure similar to Auer rods,^15^ but some inclusions were more voluminous and irregularly shaped, and were called “giant inclusions” or “megagranules.”^16^

Auer bodies were predominantly found in APL and were characterized by activity for Sudan black B and the PAS reaction cytochemically. It was thought that Auer bodies resulted from the fusion of primary granules in promyeloblasts based on their shared cytochemical reactions.^17^ Ultrastructural investigation demonstrated that Auer bodies were large membrane-bound organelles with a crystalline core, although some of them exhibited a more lamellar texture.^9,18^ The above descriptions of Auer bodies were consistent with findings in the present study based on cytochemistry and ultrastructure.

Giant inclusions were often demonstrated in neutrophils from patients with the Chediak-Higashi syndrome (CHS), occasionally found in acute myelomonocytic leukemia. These giant granules contained heterogeneous deposits and filamentous materials, and were thought to result from the fusion of primary and secondary granules based on their activity of myeloperoxidase under pathologic conditions.^19,20^

In this study, the homogeneous dense matrix, the positivity for myeloperoxidase, and the surrounding lace-like dense membrane mark them as different from the giant granules in CHS. The dense lace-like membrane associated with giant inclusions in our study was continuous with rER profiles nearby, some of which looked like the double-membrane-limited lysosomes found in CHS monocytes by combined electron microscopy and ultrastructural cytochemistry.^21^

An ultrastructural study demonstrated that prominent dilated rER, multi-laminar rER and complex stellate arrangements of rER appeared to be morpho-genetically related in APL.^22^ In this study, all the giant inclusions, Auer bodies, rER, and primary granule were characterized by high electron density and activity of myeloperoxidase of APL. This suggested that the giant inclusions might originate from expanded rER cisternae with abundant synthesized matrix within the cisternae. The presumption was substantiated by common myeloperoxidase activity of the giant inclusions and rER in promyeloblasts.^23^

**Figure 8.**
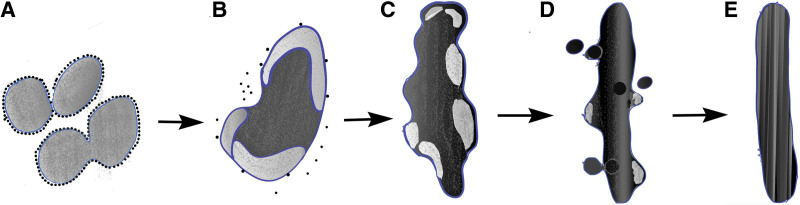
Schematic representation of the development of the Auer rod. (A) Aggregates of rER cisternae, containing a loose and lightly textured matrix, start to coalesce. (B) Continued rER cisternal fusion results in a rounded-to-oval giant inclusion. The interior is starting to condense as evidenced by a sloughing off of degenerate membrane in loops. (C) Degenerate membrane loss continues, as the giant inclusion starts to elongate. (D) A pro-Auer body, with few loops of degenerate membrane. Primary granules are budding off the surface. (E) The mature Auer body containing a crystallized interior. rER = rough endoplasmic reticulum.

Additionally, some giant inclusions showed an intermediate state between giant inclusions and Auer bodies, including a rod-like main body, furcate terminals and fewer, small membrane loops. We interpret the membrane loops as membrane being removed as the content of the body condenses and the body, as a whole, evolves from a more rounded or oval giant inclusion (asterisked in Fig. 4, for example) to a more slender and less voluminous rod-like pro-Auer body (Fig. 5). We termed these structures “pro-Auer bodies” because of the suggestion of a transition from giant inclusions to mature Auer bodies. Interestingly, in promyeloblasts, the myeloperoxidase stain demonstrated some primary granules located in the perinuclear space and within pro-Auer bodies, with a few of them budding off pro-Auer bodies. It suggested the possibility that Auer bodies originated from expanded rER cisternae rather than from the fusion of primary granules (Fig. 8). However, this presumption is contradicted by the idea in the literature that Auer bodies result from the fusion of cytoplasmic granules in promyeloblasts,^2,10^ and the novel hypothesis requires further identified using advanced devices.

ER is a dynamic membrane and serves many roles, including calcium storage, protein synthesis, transport and folding, lipid and steroid synthesis, and carbohydrate metabolism.^24^ Performing above diverse functions requires such distinct domains of ER with different architectures as tubules, sheets, and nuclear envelope.^25,26^ These structures are consisted with variant morphologies of Auer bodies and giant inclusions in this study, although transformation processes of giant inclusions associated with ER and rER to Auer bodies need to be demonstrated by dynamic techniques such as super-resolution microscopy and 3D correlative fluorescence and electron microscopy that developed in recent years.^27^

It is also possible that there is a morphological heterogeneity to Auer bodies, reflecting abnormalities that are almost a hallmark of the neoplastic process. One aspect of their development relates to how the crystalline component arises. This remains to be addressed in future work, although our preliminary findings include crystalline inclusions found within some rER cisternae; in a process as not yet defined, these rER cisternae may enter into the developmental process of Auer rod formation as illustrated in our Figure 7.

## 5. CONCLUSION

This study suggests the novel idea that expanded rER cisternae transform into giant inclusions and then Auer bodies partly as a result of the intracisternal accumulation of compounds rich in peroxides, and the progressive condensation of internal material by the release of loop-like membranous elements; further, that primary granules were directly released from pro-Auer bodies in a process which bypasses the Golgi apparatus in promyeloblasts of APL.
